# Lemon-derived nanovesicles facilitate trans-kingdom transfer of lncRNAs to human cells

**DOI:** 10.3389/fmolb.2025.1697575

**Published:** 2025-11-17

**Authors:** Vincenza Tinnirello, Roberta Gasparro, Giulia Duca, Laura Miozzi, Silvia Rotunno, Francesco Mercati, Alice Conigliaro, Riccardo Alessandro, Stefania Raimondo

**Affiliations:** 1 Department of Biomedicine, Neurosciences and Advanced Diagnostics (Bi.N.D), University of Palermo, Section of Biology and Genetics, Palermo, Italy; 2 Institute for Sustainable Plant Protection, National Research Council of Italy (IPSP-CNR), Torino, Italy; 3 National Research Council, Institute of Biosciences and Bioresources (IBBR), Palermo, Italy; 4 Navhetec s.r.l., Spinoff of the University of Palermo, Palermo, Italy; 5 ATeN (Advanced Technologies Network) Center, University of Palermo, Palermo, Italy; 6 Institute for Biomedical Research and Innovation (IRIB), National Research Council (CNR), Palermo, Italy

**Keywords:** plant-derived nanovesicles (PDNVs), lemon nanovesicles (LNVs), long noncoding RNAs (lncRNAs), cross-kingdom regulation, miRNA sponging

## Abstract

Plant-derived nanovesicles (PDNVs) are emerging as a novel class of biological messengers, capable of crossing biological barriers and transferring bioactive molecules to human cells. We have previously isolated and characterized nanovesicles from lemon juice (LNVs) that interact with human cells, modulating the mechanisms of oxidative stress and inflammation. However, the mechanisms through which LNVs exert their effect are still poorly understood. Extensive researches have been conducted on the microRNA content of PDNVs, however the profile and role of long noncoding RNAs (lncRNAs) within the vesicles remain unexplored still. In the present study, the lncRNA cargo of LNVs and its role was investigated; highly conserved lncRNAs among *Citrus* species was highlighted, with a notable enrichment of LM_XLOC_013494 within the vesicles. The lncRNA was successfully transferred to human hepatic (THLE-2) and intestinal (CACO-2) cells treated with LNVs, as confirmed by RT-qPCR and RNA *in situ* hybridization. Bioinformatic prediction analyses coupled with experimental validation revealed that the isolated lncRNA acts as a molecular sponge, specifically targeting human miR-181b-3p and miR-4420. Importantly, LNV-treated cells showed a statistically significant downregulation of these miRNAs (p ≤ 0.05), suggesting a cross-kingdom regulatory role for the plant lncRNA in modulating human gene expression. Overall, to our knowledge, this study provides novel insights into the trans-kingdom transfer of plant-derived lncRNAs, expanding upon previous findings and offering new experimental evidence.

## Graphical Abstract

**Figure:**
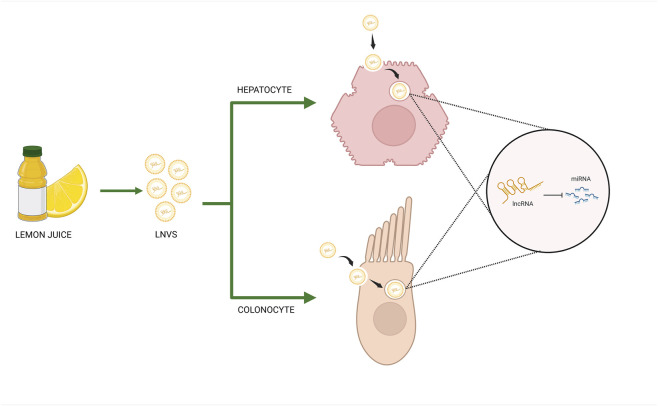
Lemon-derived nanovesicles (LNVs) deliver long noncoding RNAs (lncRNAs) into human hepatic and intestinal cells, enabling cross-kingdom regulation of gene expression via miRNA sponging mechanism (Created in BioRender. RAIMONDO, S. (2025) https://BioRender.com/yhwk2aq).

## 1 Introduction

Extracellular vesicles (EVs) represent a pivotal system of intercellular and trans-kingdom communication, orchestrating the transfer of biological signals between cells (van Niel et al., 2018; Raposo and Stahl, 2019). While the molecular mechanisms underlying selective packaging and targeted delivery of EV cargo are not fully understood, there is increasing evidence that EV cargo, consisting of a wide array of biomolecules, plays a central role in determining EV’s functional properties (Dixson et al., 2023; Mir and Goettsch, 2020). In the frame of increasing interest in EVs, plant-derived nanovesicles have recently emerged as a class of biological vesicles, exhibiting promising potential in human health applications. PDNVs, isolated from different plant species, are characterized by high biocompatibility, structural stability, and the ability to cross biological barriers (Lian et al., 2022). Emerging evidences indicate that PDNVs show several bioactive properties, including anti-inflammatory (Ye et al., 2024; Urzì et al., 2023), immunomodulatory (Nguye et al., 2023), and antioxidant activities (Perut et al., 2021). These functional characteristics suggest a potential role of PDNVs in modulating cellular physiology and signaling pathways in mammalian systems. However, despite the encouraging findings, the molecular mechanisms underlying the beneficial effects of PDNVs remain often unclear. The biomolecules encapsulated and protected by the vesicles are thought to play a crucial role in mediating these effects (Pocsfalvi et al., 2018). Among them, non-coding RNAs (ncRNAs) have emerged as critical regulators of cellular processes. They are involved in the regulation mechanisms of gene expression at multiple levels, including chromatin remodeling as well as transcriptional and post-transcriptional modifications (Panni et al., 2020; Mattick and Makunin, 2006). Among ncRNAs, long non-coding RNAs (lncRNAs) have attracted significant interest due to their complex regulatory role in important biological processes (Statello et al., 2021). These molecules, with a length of more than 200 nucleotides, can be transcribed from exons, introns, intergenic regions or 5′/3′untranslated regions and fold into intricate secondary structures that facilitate their interactions with DNA, RNA and proteins (Gut et al., 2012; Batista and Chang, 2013). LncRNAs regulate gene expression through multiple mechanisms. Indeed, they can act as molecular sponges or decoys for miRNAs, preventing degradation of target mRNA. Additionally, specific lncRNAs can modulate transcription factors, enabling their binding to promoters and thereby regulating the transcriptional regulation of target genes (Yan and Bu, 2021).

Based on these premises, we hypothesized that the beneficial effects of PDNVs may partially be attributed to the presence of specific lncRNAs. Given our extensive experience in the lemon-derived nanovesicles (LNVs) (Urzì et al., 2023; Raimondo et al., 2015; Raimondo et al., 2022), in the present work we focused our efforts to: (i) characterize the lncRNA cargo of LNVs, (ii) investigate whether these lncRNAs can be transferred to human cells, and (iii) assess their potential regulatory function in human cells. This work addresses a critical gap in the current understanding of PDNV-mediated interspecies communication and creates new opportunities for the therapeutic exploitation of plant-derived nanovesicles.

## 2 Materials and methods

### 2.1 LNV isolation

Lemon-derived nanovesicles (LNVs) were isolated from the juice of *Citrus limon* L. according to procedures previously described (Raimondo et al., 2015). The fruits, sourced from a private farmer, were thoroughly washed with water and manually squeezed. The juice obtained was subjected to two sequential centrifugations at 3,000 × g for 15 min, followed by two centrifugations at 10,000 × g for 30 min. The supernatant was filtered with 0.8 μm pore size membranes, then centrifuged at 16,500 × g for 1 h and again subjected to a filtration step with 0.45 μm pore size membranes, then centrifuged at 16,500 × g for 3 h. Finally, the resulting supernatant was subjected to a final ultracentrifugation at 100,000 × g for 1 h 45 min using a fixed-angle rotor (Type 70 Ti). The obtained pellet was washed and suspended in phosphate-buffered saline (PBS) (Raimondo et al., 2015). The isolated vesicles have been previously characterized by our research group at the proteomic, metabolomic, morphological, and size distribution levels, ensuring the reproducibility and quality of the preparations used in this study (Urzì et al., 2023; Raimondo et al., 2015; Raimondo et al., 2022).

### 2.2 Cell culture

The THLE-2 cell line (ATCC CRL-2706™, LGC Standards, Manassas, VA, USA) was used as an *in vitro* model of healthy human hepatocytes. Cells were cultured on a coating of 0.03 mg/mL bovine collagen type I (Advanced Biomatrix, San Diego, CA, USA) and 0.01 mg/mL bovine serum albumin (Sigma-Aldrich, St. Louis, MO, USA). The culture medium consisted of RPMI 1640 (Euroclone, UK) supplemented with 10% fetal bovine serum (FBS, Euroclone, UK), 1% penicillin (100 U/mL) and streptomycin (100 μg/mL), as well as 0.3 mL recombinant human epidermal growth factor (EGF, 10 μg/mL) and 0.4 mL phosphoethanolamine (PEA, 100 μg/mL). CACO-2 cell line (ATCC HTB-37™, LGC Standards, Manassas, VA, USA) was used as an *in vitro* model of human enterocytes. Cells were maintained in Eagle’s Minimum Essential Medium (EMEM) (ATCC, Manassas, VA, USA) supplemented with 20% fetal bovine serum (FBS, Euroclone, UK), 1% penicillin (100 U/mL), and streptomycin (100 μg/mL). Cell cultures were incubated in a humidified atmosphere of 5% CO_2_ at 37 °C.

### 2.3 RNA purification and cDNA synthesis

Total RNA was purified from lemon juice, LNVs, and cell lines.

#### 2.3.1 RNA purification from lemon juice and LNVs and RNase protection assays

RNA was isolated from the previously lyophilized lemon juice. To extract only RNAs located inside EVs, an RNase protection assay was carried out following the procedure described in Karimi et al. (Zand Karimi et al., 2022), using RNase A (Qiagen, Hilden, Germany; diluted in 15-mM NaCl, 10-mM Tris–HCl pH 7.5) with a final concentration of 5 μg/mL and an incubation step at room temperature (RT) for 30 min. Immediately after RNase A treatment, 0.5 mL of cold (4 °C) PureLink™ Plant RNA Reagent (Thermo Fisher Scientific, Waltham, MA, USA) was added to 100 µL of the mixture. The solution was mixed by vortex until the sample was thoroughly resuspended, followed by 5 min of incubation at room temperature (RT) and then centrifuged at 12,000 x g for 2 min at RT. The supernatant was transferred to a clean RNase-free tube, adding 0.1 mL of 5 M NaCl to the clarified extract. The solution was then shaken by tapping, followed by the addition of 0.3 mL of chloroform, mixed thoroughly by inverting the tube for 30 s, and then centrifuged at 12,000 x g for 10 min at 4 °C. The aqueous phase was recovered and mixed with an equal volume of cold isopropyl alcohol to precipitate the RNA and let stand at room temperature for 10 min. RNA pellet was washed using cold 75% ethanol and then, after centrifugation at 12,000 x g for 1 min at RT, the supernatant was carefully decanted. RNA was finally resuspended in 30 µL of RNase-free Water. To inhibit RNase A activity, used in the preliminary step, a mixture of 10 mg/mL RNase Inhibitor (Thermo Fisher Scientific, Waltham, MA, USA) and 40 units/mL of RNase Out (Invitrogen, Waltham, MA, USA) was added to the extracted RNAs, which were stored at −80 °C until use. The RNA quality and quantity were assessed using either a ThermoFisher NanoDrop One spectrophotometer and an Agilent 2100 Bioanalyzer Instrument. Total RNA was subsequently reverse transcribed into cDNA using the High-Capacity cDNA Reverse Transcription kit (Applied Biosystems, Foster City, CA, USA).

#### 2.3.2 RNA isolation from human cell lines

THLE-2 and CACO-2 cells were seeded in 12-well plates at a density of 100,000 cells per well. 24 hours after seeding, cells were treated with two different LNVs concentrations, 25 and 50 μg/mL respectively, and incubated at different time points (6, 24, 48 h). At the end of each treatment, RNA was isolated using the commercially available Nucleospin miRNA Kit (Macherey-Nagel, Düren, Germany), which enables the isolation of both total and microRNA, according to the manufacturer’s instructions. The concentration and purity of extracted RNA were determined by a Nanodrop spectrophotometer (NanoDrop Technologies, USA). Total RNA was subsequently reverse transcribed into cDNA using the High-Capacity cDNA Reverse Transcription kit (Applied Biosystems, Foster City, CA, USA). The miRNAs of interest were reverse transcribed using the TaqMan™ microRNA reverse transcription kit (Applied Biosystems™, USA) employing specific primers for U6, and mir-4420.

### 2.4 Polymerase chain reaction (PCR)

Total cDNA was used to amplify the specific plant lncRNA LM_XLOC_013494 and the human lncRNA H19 by polymerase chain reaction (PCR). Primer sequences are reported in Table 1. The PCR amplification reaction was conducted, using yourSial Taq mix kit (Sial Group, Italy) and following the instructions provided by the kit. The reaction mixture for LM_XLOC_013494 was initially heated at 95 °C for 1 min, followed by 40 cycles of denaturation at 95 °C for 15 s, annealing at 60 °C for 15 s, and extension at 72 °C for 120s. The reaction mixture for H19 was initially heated at 95 °C for 1 min, followed by 40 cycles of denaturation at 95 °C for 15 s, annealing at 58 °C for 15 s, and extension at 72 °C for 60s. After amplification, the PCR products were subjected to electrophoresis on a 2.5% (LM_XLOC_013494) and 1.5% (H19) agarose gels and visualized by Chemidoc acquisition instrument (Bio-Rad, USA).

**TABLE 1:** Primer sequences used in PCR and Real-Time PCR.

Primers	Forward	Reverse
LM_XLOC_013494	TCTGTGAGGCGTGAATGAG	AGTAGCCGTCAAGCACAAG
H19	AAGGAGCACCTTGGACATCTG	AGGTTTAGGGGATCGAGGGCT
ACTIN	TCCCTTGCCATCCTAAAAAGCCACCC	CTGGGCCATTCTTCCTTAGAGAGAAG

### 2.5 Real time PCR

The presence of plant lncRNAs in LNVs and human THLE-2 cells treated with LNVs was confirmed by RT-PCR (Step One Real-time PCR system, Applied Biosystem) in a 20-μL reaction containing 300 nM of each primer, 2 μL of template cDNA, and 18 μL of 2X SYBR Green I Master Mix. Actin was used as an endogenous control. Changes in lncRNA levels between control and treated samples were determined by the 2^−ΔCt^ method.

The list of primers used is reported in Table 1.

To assess the levels of miRNAs in human cell lines treated with LNVs, each RT-PCR reaction was prepared with TaqMan™ Fast Universal PCR Master Mix (Applied Biosystems™, USA). The primers used were hsa-miR-4420, has-miR-181b-30, has-miR-199a-3p and U6 snRNA (all from Applied Biosystems™, USA). The levels of miRNAs were normalized to U6 snRNA, and data were analyzed using the 2^−ΔΔCt^ method.

### 2.6 *In situ* hybridization analysis

The presence of investigated plant lncRNAs in human THLE-2 and CACO-2 cells treated with LNVs was evaluated using the BaseScope Detection Reagent Kit v2 - RED. Briefly, the treated THLE-2 and CACO-2 cells were seeded in a 8-well chamber and, after 24 h, treated with 70 μg/mL LNVs for 24 h. This concentration was selected specifically for the RNA scope assay to enhance image resolution and signal clarity during qualitative visualization of RNA molecules. At the end of treatment, cells were fixed with Neutral Buffered Formalin 10% and then dehydrated and rehydrated with ethanol scale at different concentrations. Then, a dilute protease III solution was applied for 10 min. After two washes, a probe specifically designed to bind the plant lncRNA sequence of interest was used in the cell hybridizetion for 2 h at 40 °C. Subsequently, eight amplification steps and signal detection reactions were performed according to the kit instructions, as described in the BaseScope Detection Reagent Kit v2 - RED User Manual. Finally, for better bright-field visualization, the cells were stained with Gill’s 50% hematoxylin solution and the slides mounted with Ecomount (Advanced Cell Diagnostics). To ensure the accuracy of the assay, internal positive and negative control provided by the BaseScope™ kit was included in all experiments. All images were acquired at 40× magnification.

### 2.7 Bioinformatic analysis

To identify potential homologous sequences in different species, the sequence homology of the lncRNA LM_XLOC_013494 was investigated using RNAcentral (http://rnacentral.org/), a comprehensive database of noncoding RNA sequences (The RNAcentral Consortium et al., 2017); homology was evaluated according to sequence similarity, E-value, percent identity, query and target coverage, and presence of gaps. The subcellular localization of the lncRNA LM_XLOC_013494 was predicted using lncLocator 2.0 (http://www.csbio.sjtu.edu.cn/bioinf/lncLocator2/) (Lin et al., 2021).

The bioinformatic tools miRDB (https://mirdb.org) and linc2function (https://bioinformaticslab.erc.monash.edu/linc2function) (Chen and Wang, 2020; Ramakrishnaiah et al., 2023) were used to explore the RNA interactome and predict potential miRNAs interacting with the lncRNA LM_XLOC_013494. The results of both analyses were cross-referenced, and miRNAs with the highest prediction scores in both analyses were selected for further analyses.

### 2.8 Statistical analysis

Data are reported as mean ± standard deviation (SD) of biological replicates. Statistical analysis was conducted using GraphPad Prism software (GraphPad software, Inc., La Jolla, CA). The normality of data distribution was assessed by the Shapiro-Wilk test. For normally distributed data, the statistical significance of the differences was analyzed using a two-tailed Student’s T-test; otherwise, a non-parametric method (Mann-Whitney test) was applied to compare the groups. For the statistical evaluation of miRNA levels in cells treated with LNVs, a one-sample t-test was performed. A *p-value* ≤ 0.05 was considered statistically significant. The statistical details for each experiment are provided in the corresponding figure legends.

## 3 Results

### 3.1 LncRNAs conserved in *Citrus* species are present in LNVs

**FIGURE 1:**
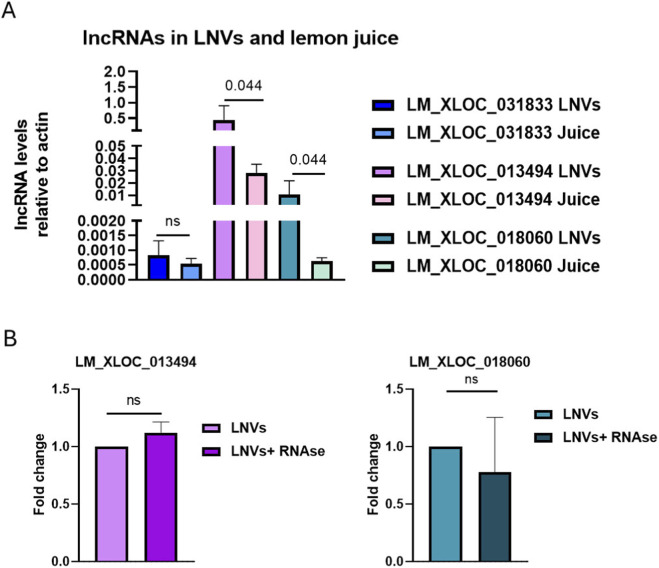
LncRNAs conserved in Citrus species are present in LNVs **(A)** RT-PCR analysis was performed to evaluate the presence and relative abundance of three lncRNAs in *Citrus limon* juice and LNVs (LM_XLOC_031833, LM_XLOC_013494, and LM_XLOC_018060). LncRNAs levels were normalized to actin. (LNV samples n = 3–8; juice samples n = 2); **(B)** RT-PCR analysis of the levels of the two most abundant lncRNAs (LM_XLOC_013494 and LM_XLOC_018060) was performed on LNVs, either untreated or treated with RNase A. LncRNA levels were normalized to actin (LNV samples n = 3; LNV + RNAse samples n = 3). Data is reported as mean ± SD. Statistical significance was assessed by a nonparametric test (Mann-Whitney test).

Through an extensive available literature (Ke et al., 2019), we identified three lncRNAs that are highly conserved in *Citrus* species: LM_XLOC_031833, LM_XLOC_013494, and LM_XLOC_018060. The presence and levels of these selected lncRNAs were evaluated in *Citrus limon* juice and LNVs isolated from it. Two out of the three lncRNAs studied - LM_XLOC_013494 and LM_XLOC_018060 - were detected in both LNVs and juice. Interestingly, a more significant abundant level (*p* ≤ 0.05) in LNVs compared to the juice was detected, suggesting an enrichment of these lncRNAs within the vesicles (Figure 1A). To confirm that these specific lncRNAs are localized within LNVs rather than being on their surface, we performed RNase A treatment on the isolated LNVs. This approach selectively degrades the external RNA while leaving the encapsulated RNA protected. Both lncRNAs remained detectable by RT-PCR after RNase A treatment, indicating that they are encapsulated within the vesicles and not simply bound to the vesicle surface (Figure 1B).

Since LM_XLOC_013494 was the most abundant among the three studied lncRNAs in our LNVs, we focused our work on this specific lncRNA. Homology search performed by RNAcentral highlighted a high degree of sequence similarity with the *Citrus sinensis* lncRNA CSIN_LNC000747. The analysis also highlighted homology, even if limited identity and coverage, with the *Homo sapiens* lncRNA HSALNT0205494 (Table 2).

**TABLE 2:** Alignment parameters and similarity metrics identified during the analysis with RNAcentral.

lncRNA	Species	E-value	Identity (%)	Query coverage (%)	Target coverage (%)	Gaps (%)
CSIN_LNC000747	*Citrus sinensis*	1.1e-63	97.75	99.63	0.41	0.00
HSALNT0205494 (chr13:94,804,713–94,827,478)	*Homo sapiens*	2.3e+2	59.18	33.96	3.37	7.14

Given the observed sequence similarity between LM_XLOC_013494 and the human lncRNA HSALNT0205494, a bioinformatic *in silico* analysis was conducted to verify that the primers designed for LM_XLOC_013494 did not recognize human sequences present in our samples. The BLAST analysis confirmed that the primer binding sites are specifically located within the plant lncRNA sequence without any overlapping in the homology region with human lncRNA, thus ensuring the specificity of our assays (data not shown). In addition, the full sequence of LM_XLOC_013494 was used to predict its localization in the different cellular compartments, including nucleus, cytoplasm, exosome, ribosome, and cytosol. The analysis performed with lncLocator predicted that LM_XLOC_013494 is predominantly localized in the nucleus with a score of 0.77 (Supplementary Figure 1).

### 3.2 THLE-2 cells treated with LNVs showed an increase in the presence of lncRNA

**FIGURE 2:**
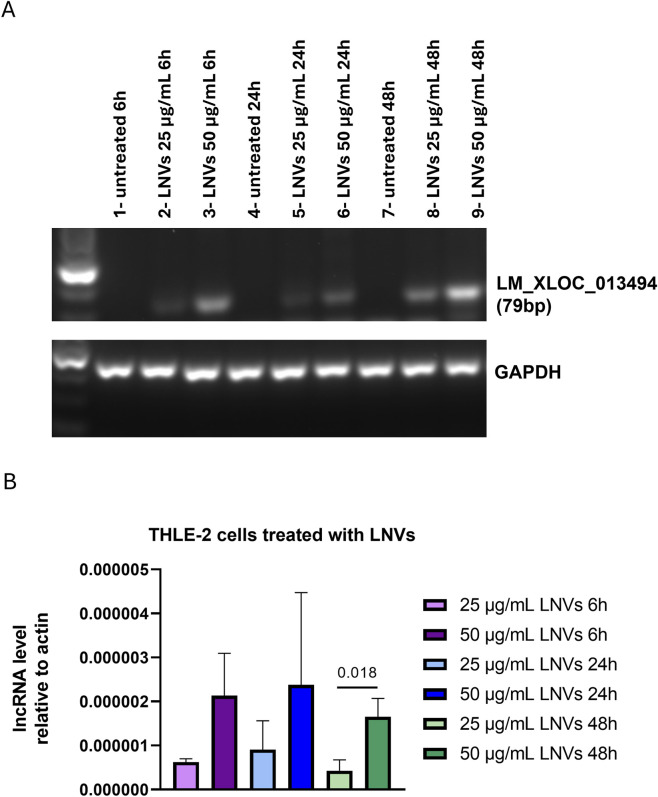
Cells treated with LNVs showed an increase in lncRNA levels. **(A)** PCR amplification of plant-derived lncRNA LM_XLOC_013494 was performed on cDNA from THLE-2 cells treated with LNVs (25 and 50 μg/mL for 6, 24 and 48 h). Amplified products were separated on a 2.5% agarose gel and visualized with a ChemiDoc imaging system. GAPDH was used as a loading control. **(B)** Levels of plant-derived lncRNA LM_XLOC_013494 were measured by RT-PCR in THLE-2 cells treated with LNVs (25 and 50 μg/mL for 6, 24, and 48 h). LncRNAs levels were normalized to actin (n = 3–6). Data is reported as mean ± SD. Statistical significance was assessed by unpaired Student’s t test (the normal data distribution was analyzed by Shapiro-Wilk test).

After the LM_XLOC_013494 identification and characterization, we evaluated whether LNVs could effectively transport and deliver this specific lncRNA to mammalian cells, thereby mediating trans-kingdom communication. Therefore, human hepatocyte THLE-2 cells were treated with two concentrations of isolated LNVs (25 and 50 μg/mL, respectively) at different time points (6, 24, and 48 h). PCR amplification of LM_XLOC_013494 in THLE-2 treated cells, followed by agarose gel electrophoresis revealed the presence of the lncRNA in LNV-treated cells (Figure 2A). To validate the specificity of the plant-derived lncRNA detection, we also assessed the expression of the human lncRNA H19 as an internal control. As shown in Supplementary Figure 2A, H19 was consistently expressed across all samples, regardless of LNV treatment.

To further confirm these findings, we performed RT-qPCR; as shown in Figure 2B, we observed an increase in LM_XLOC_013494 levels with a dose-dependent effect observed at 6, 24, and 48 h.

**FIGURE 3:**
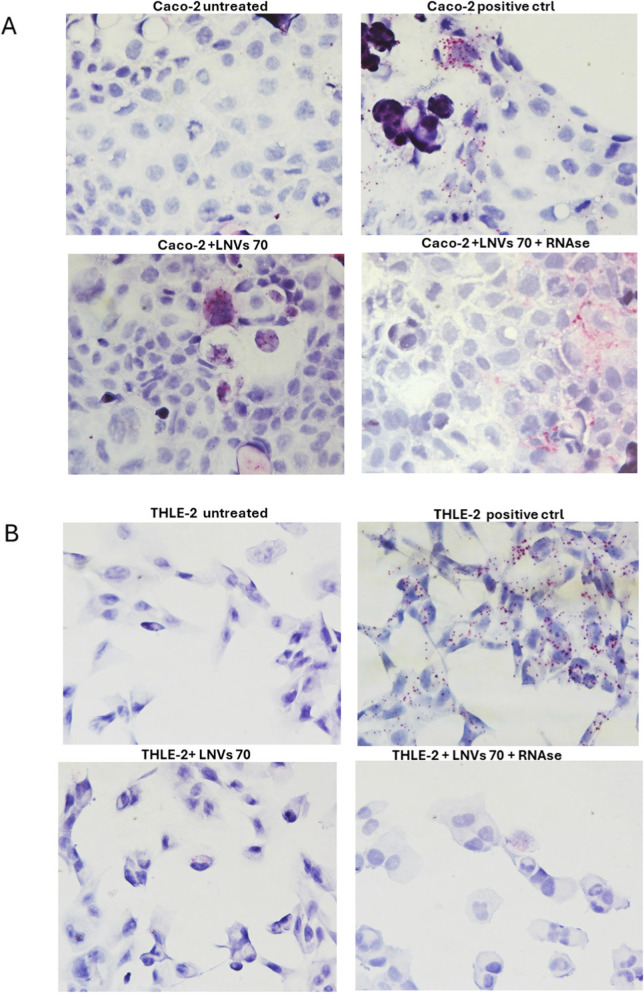
Detection of plant-derived lncRNA LM_XLOC_013494 in human cells by BaseScope™ *in situ* hybridization. **(A)** Caco-2 cells and **(B)** THLE-2 cells were treated for 24 h with 70 μg/mL of LNVs, pretreated or not with RNase A. The red/purple dotted signals indicate the intracellular presence of the plant-derived lncRNA LM_XLOC_013494. Nuclei were counterstained with hematoxylin. Internal positive controls supplied with the BaseScope™ kit were included to ensure the specificity and accuracy of the assay. All images were acquired at 40× magnification under bright-field microscopy.

Additionally, the *in situ* hybridization was performed to qualitatively detect the presence of plant-derived LM_XLOC_013494 in the human LNV-treated cells, both THLE-2 and CACO-2 cells using an additional LNVs concentration of 70 μg/mL, for 24 h. By using target-specific probes, the assay allowed to detect the lncRNA as distinct red dots in the cytoplasm of treated cells. To assess whether LM_XLOC_013494 was encapsulated within the vesicles or simply associated with their surface, the *in situ* hybridization was also carried out using LNVs pre-treated with RNase A. A marked increase in red dots underlined the presence of LM_XLOC_013494 in both THLE-2 and CACO-2 cells treated with LNVs, compared to untreated ones (Figure 3; Supplementary Figure 2B). Notably, no differences were observed between LNV-treated cells and LNV-treated samples with preliminary RNase digestion step; this finding supports the conclusion that the lncRNA is encapsulated within the plant-derived nanovesicles, thereby protected from enzymatic degradation.

### 3.3 THLE-2 and CACO-2 cells treated with LNVs showed a reduction in the level of miRNA sponged by lncRNA

**FIGURE 4:**
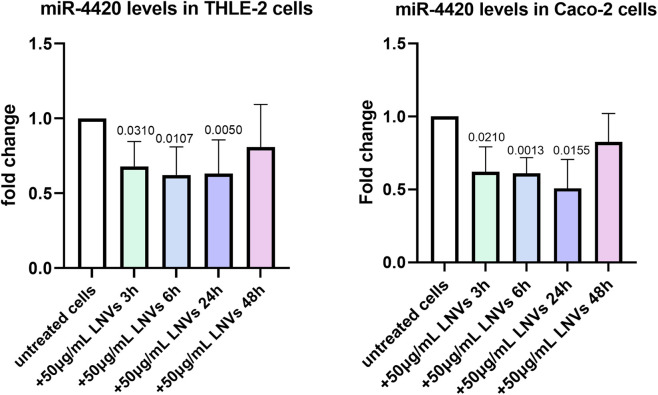
THLE-2 and CACO-2 cells treated with LNVs showed a reduction in the level of miRNA sponged by lncRNA. THLE-2 (left panel) and Caco-2 (right panel) cells were treated with 50 μg/mL of LNVs for 3, 6, 24 and 48 h. Levels of miR-4420 were measured by RT-PCR and normalized by U6 snRNA. Values are presented as fold change relative to untreated cells. THLE-2: n = 4 -6; Caco-2: n = 3 -5. Data is reported as a mean SD. Statistical significance was assessed by one-sample t-test.

In cells, lncRNAs can act as sponges of miRNAs, reducing their regulatory effect on mRNAs. To assess whether the plant-derived lncRNA LM_XLOC_013494 has functional activity after being delivered into human cells via LNVs, we first conducted a *in silico* analysis to identify miRNAs that could potentially interact with LM_XLOC_013494 based on sequence complementarity, and binding site prediction algorithms. Two bioinformatics tools, miRDB and linc2function, were employed to perform this analysis. The miRDB analysis revealed 11 possible miRNAs that could interact with LM_XLOC_013494 based on sequence complementarity and prediction of binding sites. To further explore the RNA interactome, linc2function was used, and more than 250 potential interactions were identified. The complementary approach allowed to reduce the potential targets. Indeed, both tools (Supplementary Figure 3) converged on a specific miRNA, miR-181b-2-3p, which shares the same seed sequence with miR-181b-3p and miR-4420. To validate the bioinformatic prediction and verify if the plant-derived lncRNA LM_XLOC_013494 can interact with the selected miRNAs and sponge them, the levels of miR-4420 were evaluated in both THLE-2 and CACO-2 cells, after LNVs treatment. Starting from the results described above, for this experimental step only the cells treated with 50 μg/mL LNVs, showing higher LM_XLOC_013494 transcription level, for 6, 24, and 48 h, were used. Interestingly, except at 48h, both THLE-2 and CACO-2 LNVs-treated cells showed a reduction in miR-4420 levels at each experimental time, comparing untreated ones (Figure 4). To further support the proposed sponging mechanism, we also validated the expression of miR-181b-3p, the second predicted target, which showed a reduction upon LNV treatment, consistent with the predicted interaction with the lncRNA (Supplementary Figure 4A). This findings suggested that the lncRNA LM_XLOC_013494 may function as a molecular sponge for the tested miRNAs. As a further control, the levels of a not targeted miRNA by LM_XLOC_013494 was also assessed. As expected, no modulation was observed upon LNV treatment (Supplementary Figure 4B).

## 4 Discussion

The growing interest in PDNVs has driven the scientific community to investigate the molecular mechanisms underlying their remarkable effects on mammalian cells. Although several studies demonstrated the properties of PDNVs derived from different plant sources, the specific pathways responsible for these activities remain largely undefined (Di Gioia et al., 2020).

PDNVs act as carriers of a wide range of bioactive molecules, naturally occurring in plants, encapsulated within a protective lipid bilayer. This lipid structure not only enhances the stability and bioavailability of these compounds but also facilitates their targeted delivery to recipient cells (Lian et al., 2022). In our previous studies, we demonstrated the biological properties of LNVs, highlighting their strong anti-inflammatory, antioxidant, and anti-tumor activities (Urzì et al., 2023; Raimondo et al., 2015; Gasparro et al., 2024). Based on the previous findings, the present work aimed to further investigate the molecular cargo of LNVs, with a specific focus on their non-coding RNA component. Most of the existing literature analyzing the non-coding RNA component of PDNVs focuses primarily on plant physiology or plant-microbe, and plant-parasite interactions (He et al., 2021; Zhao et al., 2024). In the context of human health, research has largely concentrated on the presence and potential roles of plant-derived miRNAs. For instance, Xiao et al. identified several miRNAs in vesicles isolated from 11 different plant species, most of which were associated with inflammatory and tumor-related pathways (Xiao et al., 2018). An intriguing additional study demonstrated that ginger-derived extracellular vesicles are internalized by the gut microbiota and contain miRNAs capable of altering their composition, thereby influencing host physiology (Teng et al., 2018). Furthermore, Baldrich and collaborators (Baldrich et al., 2019) compared the RNA profiles of EVs isolated from the apoplastic fluid of *Arabidopsis* with those of the leaves and the EV-depleted wash fluid. This study revealed that the EVs are enriched in small RNAs ranging from 10 to 17 nucleotides in length, referred to as 'tiny RNAs’ (Baldrich et al., 2019). Limited attention has been given to other classes of non-coding RNAs, such as lncRNAs (Xiao et al., 2018; Zhu et al., 2023). This gap highlights the novelty of our study, which shifts the focus toward exploring lncRNAs within LNVs and their potential functional impact on mammalian cells, offering new insights into the molecular mechanisms underlying PDNV-mediated interspecies communication.

Our study successfully identified ncRNAs highly conserved in *Citrus* species, with LM_XLOC_013494 enriched within *Citrus limon*-derived nanovesicles (LNVs). The enrichment of these lncRNAs in LNVs compared to citrus juice suggested that the vesicles may play a role in their stabilization and targeted delivery. This evidence is in agreement to other studies showing that specific types of non-coding RNAs are preferentially packaged into extracellular vesicles (Baldrich et al., 2019; Borniego and Innes, 2023). Moreover, we demonstrated that LNVs can deliver lncRNAs into human cells in a dose-dependent manner. The successful transfer was confirmed by evaluating lncRNA levels in LNV-treated cells using both RT-qPCR and *in situ* hybridization. These findings are consistent with previous studies demonstrating the role of PDNVs in facilitating trans-kingdom communication through the horizontal transfer of functional RNAs (Teng et al., 2018; Teng et al., 2021; Potestà et al., 2020).

In addition, to demonstrating RNA delivery, our study provides strong evidence that LM_XLOC_013494 maintains functional activity within human cells. Among the predicted targets of LM_XLOC_013494, *in silico* analysis identified two homologous micro-RNAs, miR-181b-3p and miR-4420. These miRNAs are known to be involved in regulations of important biological processes. Indeed, it has been implicated in oncogenic processes (Liu et al., 2014), including tumor initiation and progression in colorectal cancer (Jiang et al., 2023), showing an upregulation in hepatocellular carcinoma, where it serves as a marker of poor prognosis (Baysal et al., 2024; Ji et al., 2009; Ji et al., 2011; Chen et al., 2024). In addition, miR-181b-3p promotes inflammation and oxidative stress in liver disease, and its inhibition has been associated with protective effects in liver injury models (Wang et al., 2021; Azar et al., 2020; Cai et al., 2019). In parallel, miR-4420 can be considered particularly interesting, as no functional information is currently available in the literature, making it a new and promising candidate for uncovering novel regulatory pathways. Due to its strong sequence similarity with miR-181b-3p, which is the better-characterized homolog, miR-4420 was selected as a representative target to validate this predicted interaction. Our results revealed a reduction levels of miR-4420 following the LNV treatment in THLE-2 and CACO-2 cells, consistent with a “sponging” mechanism. However, while the reported findings suggest an interaction between the plant lncRNA LM_XLOC_013494 and specific human miRNAs, direct evidence of physical binding was not obtained in this study.

## 5 Conclusion, limitation and future directions

Our results provide new insights into the role of plant-derived lncRNAs in interspecies and trans-kingdom communication. LM_XLOC_013494, naturally occurring in LNVs, is not only efficiently delivered into human cells but also shows functional regulatory effects by modulating human miRNA levels. While these findings support the potential of LNVs as carriers of bioactive RNA molecules, some limitation of the current study should be mentioned. First, the precise intracellular fate of the delivered lncRNA remains to be elucidated. Second, although miRNA downregulation was observed, the downstream effects on target gene expression and cellular phenotypes, were not explored. Additionally, *in vivo* studies will be essential to validate the potential of LNV-delivered lncRNAs.

Overall, this study has enhanced our understanding of the therapeutic potential of LNVs, laying the foundation for the development of innovative plant nanovesicle-based compounds for the management of human diseases.

## Data Availability

The raw data supporting the conclusions of this article will be made available by the authors, without undue reservation.
