# Supplementary material for: Lemon-derived nanovesicles facilitate trans-kingdom transfer of lncRNAs to human cells
Source: Front Mol Biosci. 2025 Nov 17;12:1697575. doi: 10.3389/fmolb.2025.1697575 (PMC12666425; doi:10.3389/fmolb.2025.1697575)
Supplement: Supplementary file 1 [file DataSheet1.pdf]

Supplementary materials

Supplementary Figure 1

**Subcellular locations**	**score**
Cytoplasm	0.190137559322
**Nucleus**	**0.770323428143**
Ribosome	0.00510226882388
Cytosol	0.00665820769915
Exosome	0.0277785360111

Supplementary Figure 1: LM_XLOC_013494 localization prediction with lncLocator (http://www.csbio.sjtu.edu.cn/cgi-bin/lncLocator.py). Bolt indicates the prediction with the highest score.

Supplementary Figure 2

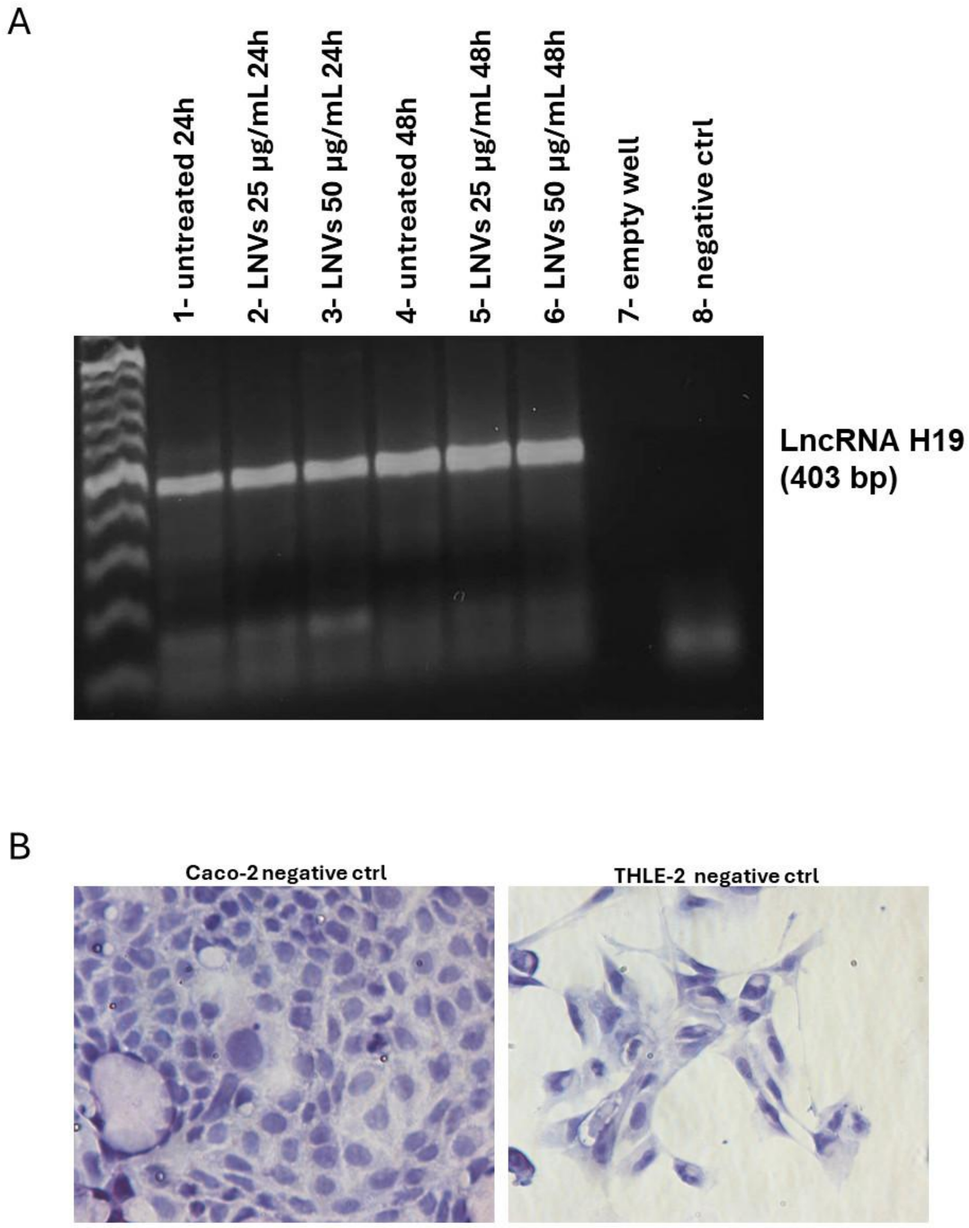

Supplementary Figure 2: A: PCR amplification of human-derived lncRNA H19 was performed on cDNA from THLE-2 cells treated with LNVs (25 and 50 µg/mL for 24 and 48 h). Amplified products were separated on a 1.5% agarose gel. **B:** Internal negative controls supplied with the BaseScope™ kit were included to ensure the specificity and accuracy of the assay. All images were acquired at 40× magnification under bright-field microscopy.

Supplementary Figure 3

A

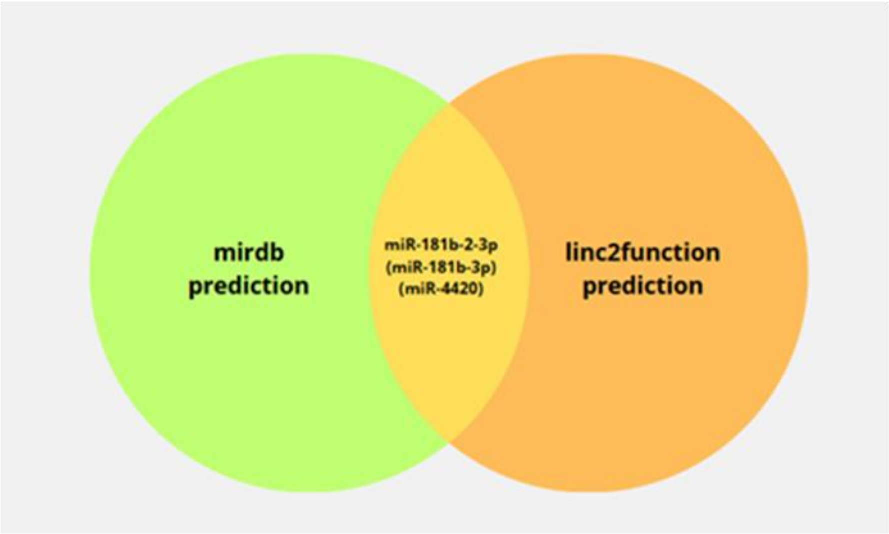

B

```
hsa-miR-181b-2-3p:    5′ - CUCACUGA--UCAAUGAAUGCA  - 3′
hsa-miR-181b-3p:      5' - CUCACUGA--ACAAUGAAUGCAA - 3′
hsa-miR-4420:         5' - GUCACUGAUGUCUGUAGCUG-AG - 3'
```

Supplementary Figure 3: Bioinformatic prediction of miRNAs interacting with the plant-derived lncRNA LM_XLOC_013494. A: Venn diagram representing the overlap between miRNAs predicted to interact with LM_XLOC_013494 using two bioinformatic tools: miRDB and linc2function. The intersection highlights the miRNAs predicted by both tools; notably, both analyses converged on miR-181b-2-3p as a common predicted target. **B:** Nucleotide sequence alignment of miR-181b-2-3p, miR-181b-3p, and miR-4420, showing conserved seed regions (highlighted).

Supplementary Figure 4

A

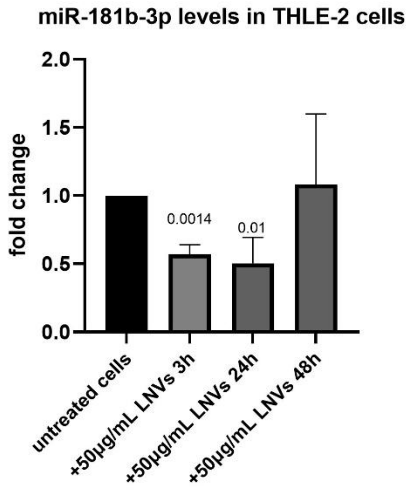

B

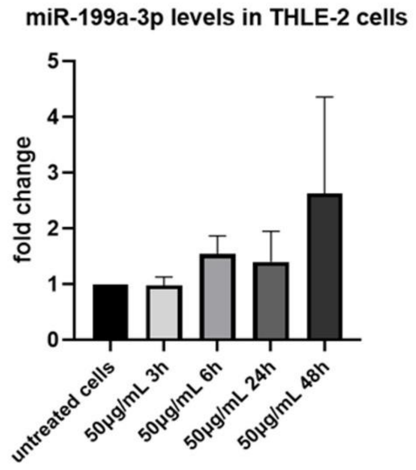

Supplementary Figure 4: A: RT-PCR analysis of miR-181b-3p in THLE-2 treated with LNVs. THLE-2 cells were treated with 50 µg/mL of LNVs for 3, 24 and 48 hours. Levels of miR-181b-3p were measured by RT-PCR and normalized by U6 snRNA. Values are presented as fold change relative to untreated cells. THLE-2: n=2-3. Data is reported as a mean SD. **B: RT-PCR analysis of miR-199a-3p in THLE-2 treated with LNVs**. THLE-2 and Caco- were treated with 50 µg/mL of LNVs for 3, 6, 24 and 48 hours. Levels of miR-199a-3p were measured by RT-PCR and normalized by U6 snRNA. Values are presented as fold change relative to untreated cells (n=2).
